# Supplementary material for: Study on distribution characteristic of tourism attractions in international cultural tourism demonstration region in South Anhui in China
Source: PLoS One. 2022 Jun 22;17(6):e0269948. doi: 10.1371/journal.pone.0269948 (PMC9216503; doi:10.1371/journal.pone.0269948)
Supplement: Attachment — Submitted filename: Response to Reviewers.docx [file pone.0269948.s002.docx]

Dear Editor

Thanks for your comments on our paper. We have revised the paper according to your comments. The following is the answers and revisions I have made in response to the reviewers’ questions and suggestions on an item by item basis.

**Reviewer 1:**

Q1: I recommend to separate the literature review part into a separate chapter.

A1: According to your opinion, the author adjusted the second paragraph of the introduction into a separate chapter. The modified part has been marked in red font.

Q2: A stronger focus should be set on the context and reasons, importance of the research and on the research questions. Literature review chapter is completely missing in this paper; I recommend to write a separate literature review chapter where the relevant international sources (preferably from international ranked and indexed journals) are processed and analysed critically and in a comparative way.

A2: According to your opinion, the author adjusted the structure of the introduction. The introduction was elaborated in four paragraphs. The first paragraph focuses on the background and reasons for choosing the topic and the significance of the research, the second paragraph is adjusted to a separate literature review chapter. The author read the journals from international rankings and indexes carefully to understand the research history and current situation of related issues. The third paragraph summarizes the existing research results, focusing on the shortcomings of the existing research results. The author hopes to make up for the shortcomings through their own research. The fourth paragraph explains the reasons for the research site selected for this study and proposed research questions. The modified part has been marked in red font.

Q3: However, the discussion part is missing. Conclusions part is appropriate and based on the results. The conclusions are very useful for the practice and policy decision makers as well, after publishing of this paper it should be promoted to them.

A3: According to your opinion, the author added a discussion part at the end of the article, discussing the three aspects of policy recommendations, research deficiencies, and future research directions. The modified part has been marked in red font.

**Reviewer 2:**

Q1: Abstract should be extended with the aims and research methods of the paper.

A1: According to your opinion, the author expanded the purpose and research methods of the abstract part of the paper. The modified part has been marked in red font.

Q2: The extension of the literature review with some more up-to-date items is recommended.

A2: According to your opinion, the author read the journals from the international rankings and indexes carefully, and applied it to the second paragraph of the introduction in the first part, expanding the literature review. The modified part has been marked in red font.

Q3: Some suggestions for the future are also recommended.

A3: According to your opinion, the author added a discussion section at the end of the article, and added suggestions for the future in the first paragraph of the discussion section.

We would like to express our gratitude again to you and reviewers for the comments on our paper. I am looking forward to hearing from you.

Yours sincerely,

Pengfei Wang
