# Supplementary material for: Study on distribution characteristic of tourism attractions in international cultural tourism demonstration region in South Anhui in China
Source: PLoS One. 2022 Jun 22;17(6):e0269948. doi: 10.1371/journal.pone.0269948 (PMC9216503; doi:10.1371/journal.pone.0269948)
Supplement: Attachment — Submitted filename: Response to Reviewers.docx [file pone.0269948.s003.docx]

Dear Editor

Thanks for your comments on our paper. We have revised the paper according to your comments. The following is the answers and revisions I have made in response to the reviewers’ questions and suggestions on an item by item basis.

**Reviewer 2:**

Q1: The article covers a current topic with appropriate methodology and excellent new results. I suggest expanding your literature review.

A1: According to your opinion, the author has expanded the literature review. The modified part has been marked in red font.

**Reviewer 3:**

Thank you for your valuable comments, we will revise according to the comments made by the reviewers.

**Reviewer 4:**

Q1: The topic of is very interesting and also relevant. The elaboration and the applied methodology seem accurate. There is still a formal problem: there are no source indications under the tables in the paper. (It has to be done even in case of field research data as well!). The bibliographic review could also be broadened, in a high-ranking journal, like Plos One, it could even be more than 50 sources instead of the present 31. After the mentioned improvements were done, the paper can be recommended to be accepted and published.

A1: According to your opinion, the author has expanded the literature review and broadened the bibliographic review. For the formal problem posed the author has added source indications to all tables in the paper. The modified part has been marked in red font.

We would like to express our gratitude again to you and reviewers for the comments on our paper. I am looking forward to hearing from you.

Yours sincerely,

Pengfei Wang
